# Impact of dissolved inorganic carbon concentrations and pH on growth of the chemolithoautotrophic epsilonproteobacterium *Sulfurimonas gotlandica* GD1^T^

**DOI:** 10.1002/mbo3.153

**Published:** 2013-12-23

**Authors:** Kerstin Mammitzsch, Günter Jost, Klaus Jürgens

**Affiliations:** Sektion Biologische Meereskunde, Leibniz-Institut für Ostseeforschung WarnemündeSeestraße 15, D-18119, Rostock, Germany

**Keywords:** Chemolithoautotrophy, DIC saturation, epsilonproteobacterium, ocean acidification, pelagic redox zones, pH, sulfurimonas

## Abstract

Epsilonproteobacteria have been found globally distributed in marine anoxic/sulfidic areas mediating relevant transformations within the sulfur and nitrogen cycles. In the Baltic Sea redox zones, chemoautotrophic epsilonproteobacteria mainly belong to the *Sulfurimonas gotlandica* GD17 cluster for which recently a representative strain, *S. gotlandica* GD1^T^, could be established as a model organism. In this study, the potential effects of changes in dissolved inorganic carbon (DIC) and pH on *S. gotlandica* GD1^T^ were examined. Bacterial cell abundance within a broad range of DIC concentrations and pH values were monitored and substrate utilization was determined. The results showed that the DIC saturation concentration for achieving maximal cell numbers was already reached at 800 *μ*mol L^−1^, which is well below in situ DIC levels. The pH optimum was between 6.6 and 8.0. Within a pH range of 6.6–7.1 there was no significant difference in substrate utilization; however, at lower pH values maximum cell abundance decreased sharply and cell-specific substrate consumption increased.

## Introduction

The hypoxic areas of the oceans, that is where the oxygen concentration is less than 2 mL L^−1^, are currently increasing on a global scale (Stramma et al. 2008). The most severe form of anoxia, with fatal consequences for higher life, constitutes the development of hydrogen sulfide-containing bottom waters. These so called “dead zones” have expanded due to eutrophication in coastal ecosystem (Díaz and Rosenberg 2008) but exist also in semi-enclosed basins with reduced water circulation such as the Black Sea, Cariaco Basin, and the Baltic Sea. The Baltic Sea is one of the largest hypoxic marine systems and it is intensely influenced by anthropogenic activities (Conley et al. 2011).

At the interface between hydrogen sulfide and oxygen and/or nitrate, different groups of sulfur oxidizing bacteria play an important role in the detoxification of hydrogen sulfide (Lavik et al. 2009). Bacterial chemolithoautotrophic denitrification occurs when there is an interface between sulfide and nitrate and has been shown to be an important process for removal of both nitrogen and hydrogen sulfide (Jensen et al. 2009; Lavik et al. 2009; Grote et al. 2012; Bruckner et al. 2013). Chemoautotrophic denitrification is a widely distributed metabolic route, inter alia, carried out by members of the *β*-, *γ*-and *ε*-proteobacteria, as well as Aquificales and some other bacterial and archeal groups, which occur across a wide range of habitats (Matějů et al. 1992; Reysenbach et al. 2009; Shao et al. 2010). However, there is evidence that *ε*-proteobacteria seem to dominate this process in marine pelagic redox zones (Brettar et al. 2006; Lin et al. 2006; Grote et al. 2007), but are also widespread in hydrothermal vents (Campbell et al. 2006). In fact, these species are responsible for the majority of chemoautotrophy in the redox zones, as they carry out 70–100% of the CO_2_ fixation in the pelagic redox gradients of the Baltic and Black Seas (Grote et al. 2008; Glaubitz et al. 2009). Different groups of *ε*-proteobacteria have been found globally distributed in marine anoxic/sulfidic areas (Grote et al. 2012; Rodriguez-Mora et al. 2013) and probably are also widespread in coastal anoxic zones with a sulfide–nitrate interface. In the Baltic Sea redox zones, *ε*-proteobacteria mainly belong to the *Sulfurimonas gotlandica* GD17 cluster (Grote et al. 2007). Grote et al. (2012) recently studied a representative of this *Sulfurimonas* cluster, named *S. gotlandica* GD1^T^ (Labrenz et al. 2013), and used genomic and physiological investigations to demonstrate high-metabolic versatility and adaptations to pelagic redox zones of this strain. The bacterium is known to reduce nitrate to dinitrogen and to oxidize thiosulfate to sulfate.

Thus, this group of *ε*-proteobacteria fulfills an important ecological role in the oxic–anoxic interface of the Baltic Sea, being primarily responsible for hydrogen sulfide detoxification and nitrate removal.

Using *S. gotlandica* GD1^T^ as a model organism for this group (Labrenz et al. 2013), we were investigating abiotic and biotic factors which regulate the growth and distribution of these bacteria in the environment. Although in previous studies the utilization of different electron donors and acceptors (Grote et al. 2012; Labrenz et al. 2013) and the impact on the distribution of this group in the redox zone (Bruckner et al. 2013) was studied, we examined here the effects of dissolved inorganic carbon (DIC) concentration and pH on growth of *S. gotlandica* GD1^T^.

The important role of chemolithoautotrophic *ε*-proteobacteria in the sulfur and nitrogen cycle led us to ask whether and how these bacteria are able to cope with changes in DIC concentrations and pH which are predicted due to ocean acidification in marine environments. According to the report of the Intergovernmental Panel on Climate Change (IPCC), a decrease of 0.3 pH units is predicted by the year 2100 and a decrease of 0.77 pH units by the year 2300 (Caldeira and Wickett 2003). However, in the deeper anoxic zones of the central Baltic basins the DIC concentration is already around 2 mmol L^−1^ and the pH is 7.1 (Beldowski et al. 2010; Schneider 2011) and acidification impacts are therefore assumed to be relatively small in the environment. Only few studies have examined the impact of DIC and pH on growth of chemolithoautotrophic bacteria, mostly with focus on carbon concentrating mechanisms, and using isolates derived from hydrothermal vent habitats (e.g., Dobrinski et al. 2005; Scott and Cavanaugh 2007). Therefore, in this study we first investigated whether different DIC concentrations and pH values have an influence on growth of *S. gotlandica* GD1^T^. Second, in order to deduce single regulating factors, we examined the influence of different pH values not only for growth but also for substrate utilization.

## Material and Methods

### Cultivation

*Sulfurimonas gotlandica* GD1^T^ was grown in anoxic artificial brackish water with the following components: 95 mmol L^−1^ NaCl, 11.23 mmol L^−1^ MgCl, 2.28 mmol L^−1^ CaCl_2_, 2.03 mmol L^−1^ KCl, 10 mmol L^−1^ HEPES, 192 *μ*mol L^−1^ KBr, 91 *μ*mol L^−1^ H_3_BO_3_, 34 *μ*mol L^−1^ SrCl_2_, 91 *μ*mol L^−1^ NH_4_Cl, 9 *μ*mol L^−1^ KH_2_PO_4_, and 16 *μ*mol L^−1^ NaF. Resazurin served as the redox indicator. To remove oxygen from the medium, the deionized water used in medium preparation was boiled for at least 10 min and then purged with N_2_ for at least 45 min. After autoclaving the medium, vitamins (Balch et al. 1979), trace elements SL10 (Widdel et al. 1983), selenite, and tungstate (Widdel and Bak 1992) were added as supplements. Nitrate (1 mmol L^−1^) was added as electron acceptor and thiosulfate (1 mmol L^−1^) as electron donor. Both were prepared anaerobically and afterward autoclaved. Although hydrogen sulfide is an important substrate in situ and was shown to be utilized by this strain (Grote et al. 2012), thiosulfate provides high cell numbers as well and is better suitable for controlled experimental investigations (Grote et al. 2012; Bruckner et al. 2013). The substrate concentrations were added in saturation for *S. gotlandica* GD1^T^, allowing exponential growth for several days. As carbon source sodium bicarbonate (filter-sterilized), was provided at a concentration of 2 mmol L^−1^. Because there is an equilibrium of hydrogen carbonate, carbonate, and carbon dioxide (DIC speciation), the carbon source will be named as DIC concentration. The distribution of the DIC speciation at pH 6.5 is 70.96% hydrogen carbonate, 28.96% carbon dioxide, and 0.07% carbonate, whereas at pH 8.0 the distribution of the DIC speciation is 95.59% hydrogen carbonate, 1.23% carbon dioxide, and 3.17% carbonate.

The bacterium was grown in batch culture at 15°C in the dark and at a pressure of 2.5 bar (N_2_-atmosphere) in all experiments. To determine the DIC saturation as well as optimum pH range 250 mL bottles were used including 50 mL headspace. In the experiments to examine substrate utilization during chemolithoautotrophic growth, 600-mL bottles were used with 100-mL headspace. Bacterial cell numbers were quantified by counting DAPI (4′,6-diamidino-2-phenylindol)-stained cells by epifluorescence microscopy. The maximal cell numbers, which were reached at the end of the exponential growth phase, represent both the yield of the culture (with respect to the substrate concentrations) and the carrying capacity under the given conditions, reflecting the efficiency of using the available substrates and converting them into bacterial biomass.

### Chemical analysis

The pH was measured with a WTW microprocessor pH meter pH 3000 and a WTW SenTix 61 pH electrode and calibrated with standard buffer solutions (pH 4.01 and 6.87). All pH measurements are reported on the National Bureau of Standards (NBS) scale. The pH was measured at the beginning (marked as pH_s_) and end (marked as pH_e_) of the incubation time. After preparing the medium (including autoclaving and cooling) and adding the substrates and the desired DIC concentration, a 20-mL subsample was taken from the anoxic medium and its pH adjusted to the desired value by addition of 0.1 mol L^−1^ hydrochloric acid at room temperature. The corresponding amount of 1mol L^−1^ HCl was then calculated and added to the medium, which was then inoculated with the bacteria. Changes in pH caused by different temperatures between the measurement and incubation temperature were calculated with the carbonate equilibration model, CO2SYS (Lewis and Wallace 1998). Nitrate was quantified colorimetrically at a wavelength of 540 nm according to the spongy cadmium method, as described by Jones (1984). Sulfate was determined turbidimetrically by Ba precipitation in a procedure modified from that of Tabatabai (1974). Here, to avoid the formation and precipitation of thiosulfate-derived zero-valent sulfur, the samples were not acidified by citric acid. Thiosulfate was analyzed with a modified method according to Zopfi et al. (2004). The samples were derivatized with 3-(bromomethyl)-2,5,6-trimethyl-1*H*,7*H*-pyrazolo[1,2-*a*]pyrazole-1,7-dione (also known as (mono)bromobimane) and then measured by HPLC (Merck), consisting of a LiChrosphere 60RP select B column (125 × 4 mm, 5 *μ*m). The eluents were 0.25% acetic acid (v/v) and HPLC-grade methanol. The methanol gradient was established as follows: 0 min: 0%, 1 min: 8%, 4.5 min: 10%, 7 min: 32%, 11 min: 32%, 18 min: 50%, 22 min: 100%, 24 min: 100%, 25 min: 0%, and 30 min: 0%. Thiosulfate was detected by a fluorescence detector (excitation: 380 nm, emission: 480 nm). Standards and reagent blanks were prepared in N_2_-purged deionized water and analyzed as described for the samples.

### Experimental design

#### Estimation of DIC saturation for growth

*Sulfurimonas gotlandica* GD1^T^ was grown in batch culture at DIC concentrations ranging from 20 *μ*mol L^−1^ to 2000 *μ*mol L^−1^ and at a pH_s_ between 7.0 and 7.5. DIC concentration was not measured directly but instead sodium bicarbonate was dissolved and then added to the medium to obtain the desired final concentration. From the theoretical equilibrium between CO_2_ compounds in the medium and in the headspace it was calculated that a maximum of 1.8% of the DIC was converted into CO_2_ gas in the headspace. Previous experiments had shown that during growth in batch culture and under the conditions applied, *S. gotlandica* GD1^T^ reaches stationary phase after 10–14 days (Grote et al. 2012; Bruckner et al. 2013). Thus, final cell concentrations at this time represent the carrying capacity for this strain under the given conditions. Therefore, we took samples after 14 days and quantified cell numbers by DAPI staining. Cell number at the beginning of the incubation time was 2.0 × 10^5^ cells mL^−1^.

The parameters for bacterial growth in relation to the DIC concentration were estimated by a nonlinear regression according to the function *B* = *B*_max_ × ([DIC]−S)/(*K*_d_ + [DIC]−S) using dynamic fitting procedure by SigmaPlot 10.0 (Systat Software, Inc., San Jose, CA), where *B* = cell abundance (cells mL^−1^), *B*_max_ = maximum cell abundance (cells mL^−1^), [DIC] = dissolved inorganic carbon concentration (*μ*mol L^−1^), *S* = threshold (*μ*mol L^−1^) and *K*_d_ = half-saturation concentration (*μ*mol L^−1^).

#### Effects of different pH values on chemolithoautotrophic growth

To identify the pH range allowing the chemolithoautotrophic growth of *S. gotlandica* GD1^T^, the bacteria were cultivated within a pH_s_ range of 6–9. Accordingly, a pH_s_ range ±0.05 of the target pH_s_ was established. HEPES (10 mmol L^−1^) was used as the buffer based on its optimum buffering capacity between pH 6.8 and 8.0. The pH of the bacterial preculture medium was between 7.0 and 7.5. Bacteria were grown in batch culture for 14 days, with the final cell number determined as described above. At the end of the incubation, the pH_e_ was controlled using the same methods described above. Cell number at the beginning of the incubation time was 2.5 × 10^5^ cells mL^−1^.

#### Substrate utilization during chemolithoautotrophic growth

After the pH range had been determined, which is suitable for chemolithoautotrophic growth of *S. gotlandica* GD1^T^, substrate utilization was investigated at selected pH values within this range. *S. gotlandica* GD1^T^ was grown under the same conditions as above, and the pH was measured at the beginning (pH_s_) and end (pH_e_) of the experiments. The experiments were conducted at pH_s_ of 7.1 (the pH of the Baltic Sea redox zones), and at pH_s_ 6.6 (the critical point at which the influence of pH on maximal cell numbers became visible before). Cell number at the beginning of the incubation time was 2.7 × 10^5^ cells mL^−1^.

Cell numbers, nitrate and thiosulfate consumption, and sulfate production were quantified daily for 14 days, although substrate utilization per bacterium was calculated only during late exponential growth (day 6–9). Nitrate was analyzed in 1 mL samples diluted 1:100, and sulfate in undiluted 1 mL samples. Thiosulfate was measured in 25-*μ*L samples centrifuged and diluted 1:10 prior to derivatization with 50 *μ*L of Monobromobimane-HEPES-EDTA-buffer. The derivative was diluted again 1:10 to obtain a thiosulfate concentration below 20 *μ*mol L^−1^, that is within the concentration range yielding the best linear relationship. Sulfate was measured immediately, whereas nitrate and thiosulfate samples were stored at −20°C until analysis. All vials used for the analyses were flushed with N_2_ to remove oxygen and to maintain the samples as oxygen-free as possible. Negative controls without bacteria had been previously performed and revealed that purely chemical reactions can be ruled out for changes in substrate concentrations at different pH values(Bruckner et al. 2013).

Statistical tests were performed using an analysis of variance (ANOVA) and an error probability of 5% followed by a post hoc test (Tukey).

## Results

### Estimation of DIC saturation for growth

**Figure 1:**
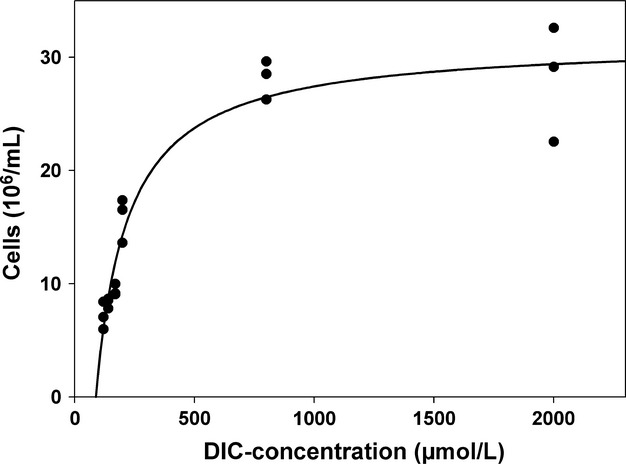
Maximal cell numbers of *Sulfurimonas gotlandica* GD1^T^ under different DIC conditions. The three replicates at each DIC concentration are shown separately. The bacterium was grown for 14 days in batch culture. Data are shown as a rectangular curve (*r*^2^ = 0.96), corresponding to a half-saturation concentration of 132.6 *μ*mol L^−1^ and a threshold concentration of 87.5 *μ*mol L^−1^ DIC.

*Sulfurimonas gotlandica* GD1^T^ grew at the whole DIC concentrations but final cell numbers increased with increasing DIC concentration up to a saturation level at around 800 *μ*mol L^−1^ DIC. At this concentration maximal cell number of 2.8 × 10^7^ ± 3.4 × 10^6^ cells mL^−1^ was reached (Fig. 1). According to the calculated dynamic fitting procedure by SigmaPlot, half-saturation concentration in DIC for achieving maximal cell numbers was 132.6 *μ*mol L^−1^, with a threshold concentration of 87.5 *μ*mol L^−1^ DIC.

### Effects of different pH values on chemolithoautotrophic growth

**Figure 2:**
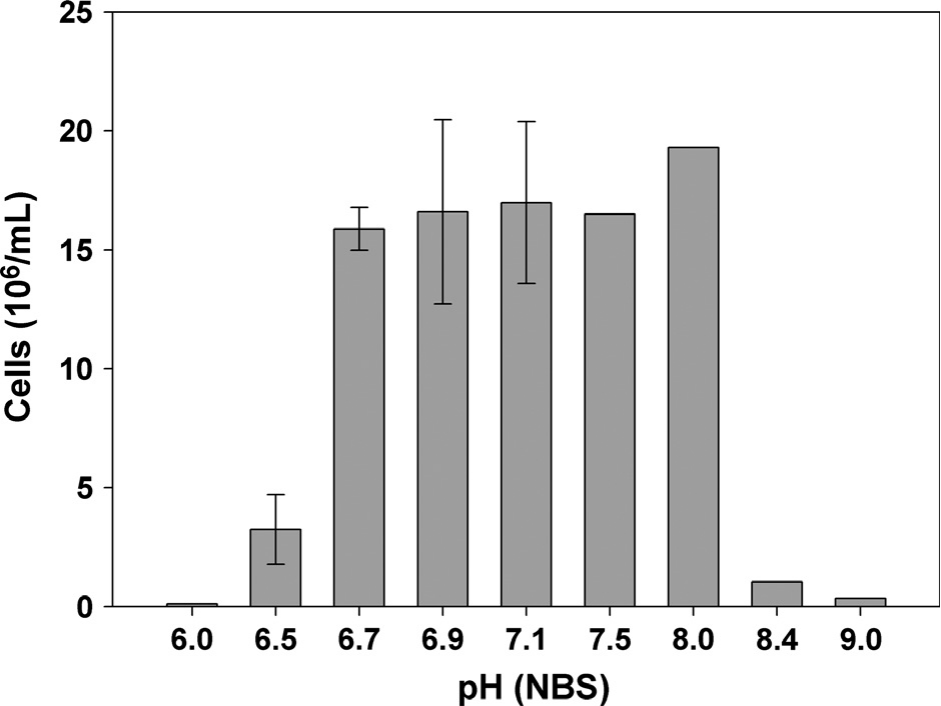
Influence of pH on maximal cell numbers of *Sulfurimonas gotlandica* GD1^T^. The bacterium was grown in batch culture at different pH values for 14 days. Values between pH 6.5 and 7.1 are the means (±SD) of three replicates. Values below and above this pH range are single data.

The optimum pH_s_ range for *S. gotlandica* GD1^T^, as judged from the final cell concentration, was between 6.7 and 8.0, with no significant differences in the maximal cell numbers of 1.7 × 10^7^ ± 2.9 × 10^6^ cells mL^−1^ (ANOVA, *P* > 0.05) (Fig. 2). At pH_s_ values above 8.0 and below 6.5, cell numbers did not differ from the initial levels, indicating that no growth occurred. At pH_s_ 6.5, bacterial cell numbers increased only slightly, resulting in a maximal cell number of 3.3 × 10^6^ ± 1.5 × 10^6^ cells mL^−1^. The pH measurements at the end of the experiment showed that the pH in the range of 6.5 and 8.4 remained constant (±0.02) during the experimental time whereas above and below these points the pH_e_ decreased by about 0.18–0.25.

### Substrate utilization during chemolithoautotrophic growth

**Figure 3:**
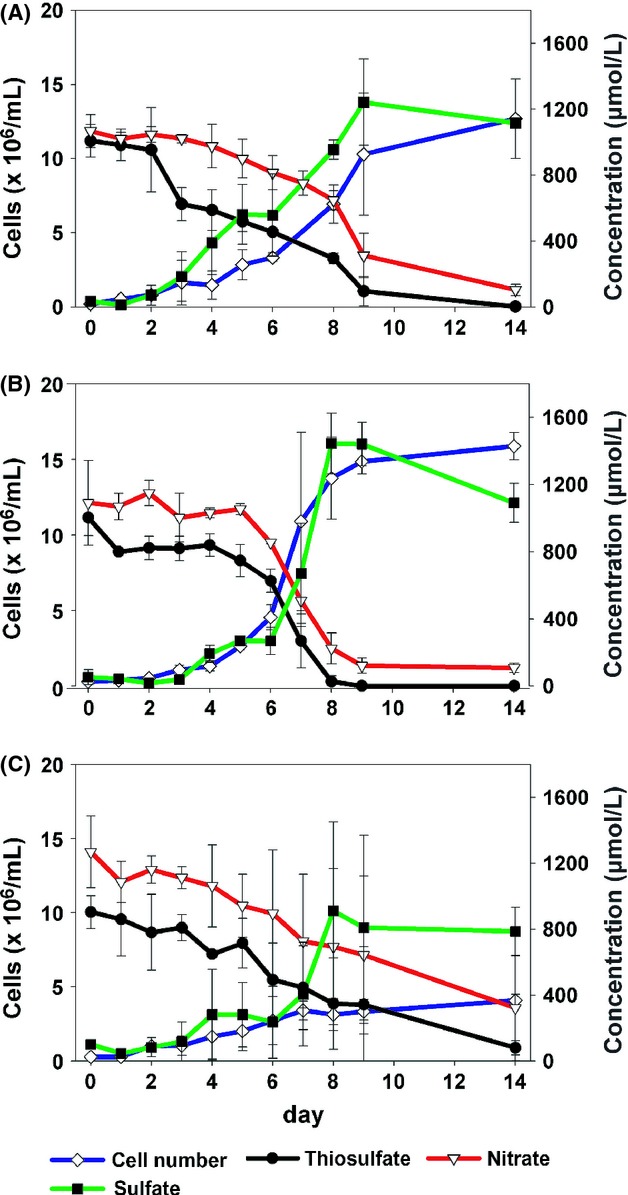
Anaerobic chemoautotrophic growth of *Sulfurimonas gotlandica* GD1^T^ in a batch culture at pH 7.1 (A), pH 6.6 (B), and pH 6.55 (C). Cell abundance and nitrate (electron acceptor), thiosulfate (electron donor), and sulfate (formed by thiosulfate oxidation) concentrations were quantified daily. (A–C) are the means of three, two, and two replicates, respectively. Error bars are standard deviations.

The aim of this study was to examine the substrate utilization of *S. gotlandica* GD1 in dependence of pH. The chosen pH_s_ values were pH_s_ 7.1 (present pH in Baltic Sea redox zones), and pH_s_ 6.6 (the critical point, at which an influence of pH on maximal cell numbers was visible). Maximal cell numbers, and substrate utilization showed no significant differences between the pH_s_ 7.1 and 6.6 values (ANOVA, *P* > 0.05) (Fig. 3). Thus, the results of pH_s_ 7.1 and 6.6 were summarized together. In all trials within these 2 pH_s_ values, a cell abundance of 1.4 × 10^7^ ± 2.7 × 10^6^ cells mL^−1^ was reached in 9 days (Fig. 3). However, at a pH_s_ directly below 6.6 cell numbers were sharply reduced (see Figs. 2, 3), for example at pH_s_ 6.55 only a maximal cell number of 3.8 × 10^6^ ± 4.1 × 10^5^ cells mL^−1^ at day 9 was achieved (Fig. 3C).

At pH 7.1 and 6.6 *S. gotlandica* GD1^T^ completely consumed the 1000 *μ*mol L^−1^ thiosulfate, metabolizing most of it to sulfate within the 9 days (1322.9 ± 201 *μ*mol L^−1^). Nitrate was only partially consumed and at the end of the experiment still 236 ± 129 *μ*mol L^−1^ of nitrate could be measured. In contrast, at pH_s_ 6.55 the bacteria used 742.5 ± 391.8 *μ*mol L^−1^ of nitrate and 824.3 ± 144.0 *μ*mol L^−1^ of thiosulfate and produced 903.8 ± 373.0 *μ*mol L^−1^ sulfate.

According to Figure 3 the exponential growth occurred between days 3 and 9. During the late exponential phase (day 5–9) differences in the substrate concentrations were the most significant, thus reducing methodological biases (which are larger when only small concentration changes occur). Hence, in the late exponential phase *S. gotlandica* GD1^T^ used 68.1 ± 12.3 fmol nitrate cell^−1^ and 43.7 ± 5.9 fmol thiosulfate cell^−1^ and produced 77.9 ± 17.9 fmol sulfate cell^−1^ at pH_s_ 7.1 and 6.6. The cellular growth rate was at both pH_s_ values around 0.4 ± 0.01 h^−1^. In contrast, at pH_s_ 6.5, the growth rate was 0.3 ± 0.02 h^−1^, but with strongly enhanced substrate turnover. In fact, the cells used 414 ± 151 fmol nitrate cell^−1^, 247 ± 209 fmol thiosulfate cell^−1^, and produced 944 ± 563 fmol sulfate cell^−1^ than cultures maintained at a higher pH.

The negative controls for all chemical analysis remained constant during the incubation time and were always in the same range as the standards samples of 0 *μ*mol thiosulfate, nitrate or sulfate, respectively, used for calibration. Hence, there was no evidence that other chemical compounds influenced the measurements. The discrepancy between thiosulfate utilization and sulfate production at 6.55 was probably caused by methodical errors. The measurements of nitrate and thiosulfate at the beginning and after 24 h confirmed the added concentrations of 1 mmol L^−1^ for each of these substrates. The pH could be kept relatively constant with a decrease of pH_e_ of 0.092 ± 0.046 units.

## Discussion

The primary aim of this study was to examine the response of the *ε*-proteobacterium *S. gotlandica* GD1^T^ toward changes in DIC and pH. This could be achieved by assessing the impact of changes in DIC concentration and pH on growth and maximal cell numbers of *S. gotlandica* GD1^T^ in batch culture growth experiments.

Hydrogen sulfide is the major substrate in anoxic waters and *S. gotlandica* GD1^T^ seems to be primarily responsible for hydrogen sulfide oxidation in the Baltic Sea (Grote et al. 2012). However, at the oxic–anoxic interface and the upper sulfidic zone thiosulfate concentrations are in a similar range as hydrogen sulfide concentrations (Bruckner et al. 2013) and thiosulfate serves as an alternative substrate for *S. gotlandica* GD1^T^. We used thiosulfate as electron donor in the experiments as substrate concentrations can be much better controlled compared to hydrogen sulfide. Although thiosulfate seemed to be entirely consumed at the end of the experiment (see Fig. 3), earlier experiments with this strain did not produce higher cell numbers with higher thiosulfate concentrations (Bruckner et al. 2013; Labrenz et al. 2013). Therefore, other potentially limiting factors, related to cell concentration, have to be considered, such as the accumulation of inhibitory metabolic products.

### Estimation of DIC saturation for growth

The growth-stimulating effects of increasing DIC concentrations for phytoplankton are well documented (e.g., Iglesias-Rodriguez et al. 2008) whereas only few studies were performed with chemolithoautotrophic bacteria. According to the current DIC concentrations of about 2 mmol L^−1^ and 3.5 mmol L^−1^ (Frey et al. 1991; Beldowski et al. 2010) in the redox zones of the Baltic and the Black Sea, our results show that these DIC concentrations are well within the range supporting maximal growth of *S. gotlandica* GD1^T^ and related epsilonproteobacteria. The results suggest that a further increase in DIC concentration in the redox zones should have no additional direct effect on these bacteria.

A higher DIC concentration in the ocean causes a shift in the DIC speciation toward carbon dioxide, resulting in a decrease of pH, that is at pH 7.1 90% of the DIC speciation is hydrogen carbonate while at pH 6.3 the balance shifts to 50% hydrogen carbonate and 50% carbon dioxide (Deffeyes 1965). However, this shift in speciation should not have an influence on growth of *S. gotlandica* GD1^T^ as a DIC concentration of 800 *μ*mol L^−1^ was already sufficient to promote maximal cell numbers (Fig. 1). Comparable saturation curves for increasing DIC concentrations had been determined for other bacterial and phytoplankton species. Clark and Flynn (2000) described the relationship between the carbon-specific growth and the DIC concentration of several marine phytoplanktons. Most of these species reached a saturation between 500 and 1000 *μ*mol L^−1^ DIC. Furthermore, Dobrinski et al. (2005) showed that for the chemolithoautotrophic *γ*-proteobacterium *Thiomicrospira crunogena*, isolated from a hydrothermal vent, the half-saturation DIC concentration was 220 *μ*mol L^−1^ and saturation was reached at 1000 *μ*mol L^−1^ DIC, which is in the same range as the values determined for *S. gotlandica* GD1^T^. In contrast, Scott and Cavanaugh (2007) showed that the chemoautotrophic *Solemya velum* symbionts reach saturation at a CO_2_ concentration of 100 *μ*mol L^−1^. However, this saturation is also well within the range of the CO_2_ concentration in the environment, where *S. velum* was collected. In addition, they could prove that these symbionts rely on CO_2_ and not on bicarbonate.

Genomic data indicate that *S. gotlandica* GD1^T^ is capable of using both CO_2_ and bicarbonate by converting intracellular bicarbonate to CO_2_ with the carbonic anhydrase (Grote et al. 2012). In addition, Dobrinski et al. (2005) could prove that the chemolithoautotrophic *γ*-proteobacterium *Thiomicrospira crunogena* has the ability to use both external CO_2_ and bicarbonate. Therefore, it seems probable that *S. gotlandica* GD1^T^ has also the ability to use both external CO_2_ and bicarbonate as inorganic carbon source.

### Effects of different pH values on chemolithoautotrophic growth

The pH range at which *S. gotlandica* GD1^T^ grew well (pH 6.6–8.0) was relatively narrow compared to that of other chemolithoautotrophic proteobacteria. For example several *γ*-proteobacterial *Thiomicrospira* species from hydrothermal vents grew at a wide pH range of 5.3–8.5 or 4.0–7.5 (Brinkhoff et al. 1999). Also for other chemoautrophic *ε*-proteobacteria a relatively wide tolerable pH range was found, for example for *Sulfurimonas paralvinellae* and *Sulfurimonas autotrophica*, the pH range was 5.4–8.6 (optimum 6.1) and 5.0–9.0 (optimum 6.5), respectively (Inagaki et al. 2003; Takai et al. 2006). *Sulfurimonas denitrificans*, the closest cultivated relative of *S. gotlandica* GD1^T^ (Grote et al. 2012), has a pH optimum of 7.0 (Timmer-ten Hoor 1975). Most of these investigated bacteria with a more extended pH range compared to *S. gotlandica* GD1 exist in habitats such as hydrothermal vents, where pH changes are frequent and rapid. Thus, the extended pH range suggests that it is an adaptation to the extremely variable conditions, whereas *S. gotlandic*a str. GD1 was isolated from a relatively stable habitat. Scott and Cavanaugh (2007) confirm this conclusion with their studies about chemoautotrophic *γ*-proteobacteria, living as endosymbionts in sulfidic/oxic interfaces. These *Solemya velum* symbionts have also a relatively narrow range of pH optimum (between pH 7.4 and 8.5), showing the same sharp decline in growth directly below and above these levels.

The substrate utilization did not differ significantly between pH_s_ 7.1 and 6.6. However, at a pH_s_ below 6.6 the situation changed drastically and growth of *S. gotlandica* GD1 was obviously impaired, and consumption of nitrate and thiosulfate was strongly reduced. Thus, the important functional role of *S. gotlandica* GD1^T^ in the redoxcline nitrogen and sulfur cycles would probably be impacted.

While intracellular pH was not measured in this study, it is supposed that the intracellular pH varies by around 0.1 units per unit change in the external pH (Hackstadt 1983). According to Booth (1985) changes in pH outside the pH optimum leads to an inhibition of both enzyme activity and cell growth (Booth 1985). However, thus far the exact mechanism of intracellular pH regulation is not completely understood. Earlier studies have shown that the regulation is energy dependent and requires a high respiratory rate (Booth 1985). Hence, the high substrate utilization per bacterium at pH_s_ 6.55 could be at least partially explained by regulation of the intracellular pH outside the optimum pH.

As substrate utilization in the batch culture experiments was significantly higher than under environmental conditions, the results rather reflect the carrying capacity of *S. gotlandica* GD1^T^ at the given conditions. Future studies should aim to more accurately simulate in situ conditions (e.g., with chemostat cultures). Due to global warming and higher CO_2_ concentrations in the atmosphere an increase in the DIC concentration and a decrease of pH by about 0.3 units in the ocean, known as ocean acidification, is predicted (Houghton et al. 2001). Our results suggest that a direct impact of acidification on *S. gotlandica* GD1^T^ and related organisms should not be very strong, as the optimum pH range for this model organism is still within the range of the predicted changes in pH. On the other hand, indirect effects on *S. gotlandica* GD1^T^ might be more important than direct ones. For example there is evidence that nitrification, the process which delivers nitrate for denitrifying bacteria, is negatively influenced by a decrease in pH and an increase in pCO_2_ (Huesemann et al. 2002; Denecke and Liebig 2003; Hutchins et al. 2009).
